# Limited Effectiveness of Carbonaceous Sorbents in Sequestering Aged Organic Contaminants in Sediments

**DOI:** 10.1021/acs.est.3c02309

**Published:** 2023-06-15

**Authors:** Allison R. Taylor, Jie Wang, Parminder Kaur, Daniel Schlenk, Jay Gan

**Affiliations:** † Department of Environmental Sciences, University of California, Riverside, California 92521, United States; ‡ College of Resources and Environmental Sciences, China Agricultural University, Beijing 100193, China

**Keywords:** carbonaceous materials, aging, bioavailability, DDT, remediation

## Abstract

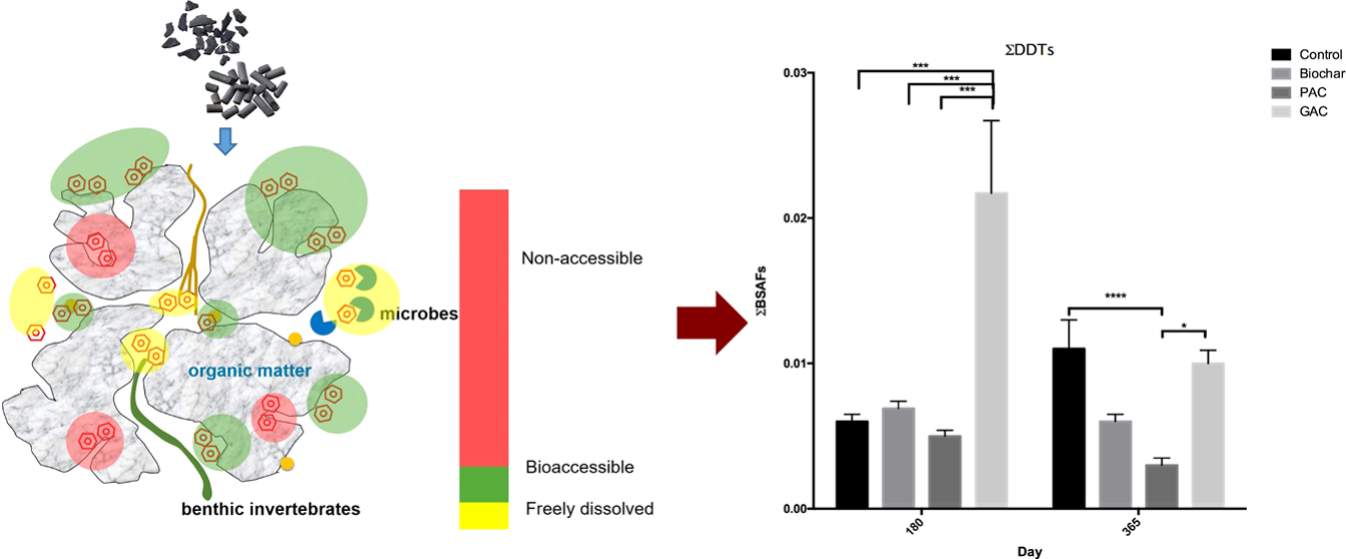

Carbonaceous materials are often proposed for use in restoring soils or sediments contaminated with hydrophobic organic contaminants (HOCs). However, the contamination of most sites is a result of historical events, where HOCs have resided in the solid compartment for many years or decades. The prolonged contact time, or aging, leads to reduced contaminant availability and likely diminished effectiveness of using sorbents. In this study, three different carbonaceous sorbents, i.e., biochars, powdered activated carbon, and granular activated carbon, were amended to a Superfund site marine sediment contaminated with DDT residues from decades ago. The amended sediments were incubated in seawater for up to 1 year, and the freely dissolved concentration (*C*_free_) and the biota-sediment accumulation factors (BSAFs) for a native polychaete (*Neanthes arenaceodentata*) were measured. Even though the bulk sediment concentrations were very high (6.4–154.9 μg/g OC), both *C*_free_ and BSAFs were very small, ranging from nd to 1.34 ng/L and from nd to 0.024, respectively. The addition of carbonaceous sorbents, even at 2% (w/w), did not consistently lead to reduced DDT bioaccumulation. The limited effectiveness of carbonaceous sorbents was attributed to the low DDT availability due to prolonged aging, highlighting the need for considering contaminant aging when using sorbents for remediation.

## Abstract 2

Carbonaceous sorbents have limited effectiveness for remediation of soil and sediment sites with aged contaminants.

## Introduction

Hydrophobic organic contaminants (HOCs), including polychlorobiphenyls (PCBs), polycyclic aromatic hydrocarbons (PAHs) and organochlorine pesticides, are found ubiquitously in soil or sediment compartments.^1−3^ However, man-made chemicals like DDT and PCBs were phased out about five decades ago in the U.S. and in many other regions. Therefore, their residues present today have, in fact, resided in the soil or sediment environment for a very long time due to their immobility and recalcitrance to degradation. During the long contact time, HOC molecules may slowly diffuse into the inner micropores or glassy regions of organic matter of soil or sediment aggregates. Recent studies have shown that sequestration, commonly termed “aging,” has resulted in irreversible sorption or reduced chemical and biological availability for such HOCs in soil or sediment.^4−7^

Amendment of carbonaceous materials has often been proposed as a remediation strategy for HOC-contaminated soils and sediments.^8−15^ Carbonaceous materials are usually the carbon that has undergone incomplete combustion through either natural (i.e., wildfires) or artificial means with biomass or fossil fuel as the starting material.^16^ Exposure to high temperatures increases both the sorption capacity and hydrophobicity of the carbon, lending to their property as a strong sorbent for HOCs.^8,13,17,18^ Activated carbon (AC) and, more recently, biochars are common carbonaceous materials that may be used to treat soil or sediment. Activated carbon is produced from either coal or biomass, “activated” by high-temperature or chemical treatments.^13^ It is most often used in a powdered form (powdered activated carbon, or PAC) that lends itself for easy use in many situations, while granular activated carbon (GAC) can be added and subsequently removed from contaminated soil or sediment (e.g., via sieving), allowing for the removal of HOCs from the contaminated site.^19^ Biochar is another type of carbonaceous sorbent, typically produced from biomass via pyrolysis at different temperatures under reduced oxygen conditions.^20,21^

The principle of carbonaceous sorbent-based remediation is based on the reduction of the labile or available fraction of HOCs through sorption or sequestration.^8,15,22,23^ Activated carbon has proven to be effective in many laboratory studies,^9,24−33^ as well as in some field trials.^10,12,34−36^ Studies have similarly shown biochars to be effective sorbents for many HOCs and metals.^37−40^ Biochars are also seen as a more sustainable alternative, as the conversion of plant biomass to biochars helps taking carbon out of the global carbon cycle.^41−44^

Although many studies have considered the effectiveness of carbonaceous sorbent amendment in sequestering HOCs in soil or sediment, to date, few studies have taken into consideration the role of contaminant aging.^45−47^ Here, we hypothesized that for aged HOCs in soil or sediment, because their availability is already reduced, addition of carbonaceous sorbents may produce a limited effect. In this study, we used the marine sediment from a Superfund site off the coast of Los Angeles to evaluate the effect of carbonaceous sorbent amendment on the bioaccumulation and availability of aged DDT residues. The findings can provide guidance on the benefit, or the lack of it, of using carbon or other sorbents for the remediation of historically contaminated sediment and soil sites.

## Materials and Methods

### Chemicals, Sediments, and Carbonaceous Sorbents

Standards of four DDT derivatives, i.e., *o*,*p*′-DDE, *p*,*p*′-DDE, *o*,*p*′-DDD, and *p*,*p*′-DDD and 4 PCB congeners (PCB 30, 67, 80, and 191) were purchased from AccuStandard (New Haven, CT). The PCBs were used as internal standards (PCB 30 and 80) or recovery surrogate standards (PCB 67 and 191). A sheet of 25-μm thin polyethylene (PE) film was purchased from BBB Accredited Business (Cleveland, OH) and was cut into 1 cm × 2 cm long strips. The PE strips were precleaned by sonication in *n*-hexane for 1 h and used for measuring the freely dissolved concentration *C*_free_. Instant Ocean salts (Blacksburg, VA) were used to make artificial seawater for all experiments. Florisil was purchased from Acros Organics (Morris Plains, NJ), and Florisil cartridges used for sample cleanup (2 g) were packed in the laboratory. A powdered activated carbon (PAC) and a granular activated carbon (GAC) were purchased from Fisher Scientific (Hampton, NH), and the biochar was purchased from Biochar Supreme (Everson, WA). Each powdered product (PAC or biochar) was sieved (no. 100 mesh, ≤0.15 mm) before use. All other chemicals and solvents were of HPLC grade or higher.

The sediment used in this study was collected from the Palos Verdes Superfund Site off the coast of Los Angeles, near the wastewater effluent outfalls (8C), where high levels of DDT residues were documented (Figure S1, in Supporting Information). Between 1947 and 1971, Montrose Chemical Company, the largest DDT manufacturer in North America at the time, discharged wastewater containing DDT into the Los Angeles County sewer system that flowed out of the White Point outfalls. An estimated 870–1450 tons of DDT was emitted and deposited onto the ocean sediment floor of the Palos Verdes Shelf.^48,49^ Consequently, approximately 44 km^2^ of sediment floor was contaminated, leading to the U.S. EPA to designate the shelf as a Superfund site in 1989.^50^ The levels of DDT derivatives remain high at present, with total sediment concentrations of DDT and its metabolites (DDE and DDD) in the range of 0.36–31.3 μg/g dry weight (dw)^51^ and the marine sediment floor continuing to act as an emission source.^52,53^

Since the DDT residues had remained in contact with the sediment for several decades, a recent study showed that the DDT derivatives exhibited remarkably reduced bioavailability, and that the reduced bioavailability was attributed to extensive contaminant aging.^6^ To simulate carbonaceous sorbent amendment in a remedial operation, the sediment was amended with 2% (w/w) of PAC, GAC, or biochar, with unamended sediment serving as the control. After the amendment, sediments were incubated, under static conditions, with a 2 cm layer of seawater for up to 12 months. Sediments were removed after 6 and 12 months and were analyzed as below. The total concentrations of DDTs in the sediment, prior to the incubation, were determined through preliminary experiments to be 23.9 ± 4.2, 154.9 ± 36.1, 6.4 ± 1.9, and 25.0 ± 13.4 μg/g OC for *o*,*p*′-DDE, *p*,*p*′-DDE, *o*,*p*′-DDD, and *p*,*p*′-DDD, respectively.

### Measurement of Freely Dissolved Concentration *C*_free_

The freely dissolved concentration *C*_free_ was measured using a method developed previously for DDTs using polyethylene (PE) film.^4,52^ Briefly, a 2.0 g dw aliquot of sediment was mixed with 2 mL of clean seawater containing 200 mg/L sodium azide (to inhibit microbial activity) in a 10 mL glass liquid scintillation vial, and one 2 cm × 1 cm strip of precleaned PE film was added to the slurry. The samples with the PE passive sampler were shaken at 120 rpm on a horizontal shaker at room temperature for 28 days. Previous experiments showed that 28 days was sufficient for the PE film to achieve partition equilibrium under the used conditions.^52^ The PE film was removed and cleaned with deionized water. Each film was then placed in a 2 mL GC vial and extracted with 1 mL of hexane via sonication for 30 min. An internal standard was added just before analysis on GC-MS. The *C*_free_ value was calculated using eq 1
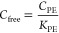
1where *C*_PE_ is the analyte concentration in the PE film and *K*_PE_ is the PE-seawater partition coefficients for a specific DDT compound derived in previous studies.^4,53^

### Bioaccumulation Assay

A marine polychaete, *Neanthes arenaceodentata*, purchased from Aquatic Toxicology Support (Bremerton, WA), was used as the exposure organism. This benthic species is native to the sediment floor of the Palos Verdes Shelf and is an important source of food for bottom-feeding fish species like the California halibut *Paralichthys californicus*.^54^ The bioaccumulation test for sediments from the Palos Verdes Shelf was modified from 28 days to 96 h to avoid ammonia toxicity and potential mortality of exposed organisms.^55^ In a 500 mL jar, 10 worms were added to approximately 100 g (dw) of sediment, and the overlaying water was refreshed daily to reduce the level of ammonia. Under the experimental conditions, DDT residues were predominantly associated with the sediment and sediment porewater, and the displacement of overlaying water (about 2 cm in thickness) was not expected to affect bioaccumulation of DDT compounds by the polychaete that dwelled mostly inside the sediment. Three replicates were used for each treatment. After 96 h, worms were removed from the sediment and placed in Petri dishes containing only seawater for 24 h to allow depuration. The worms were then frozen at −80 °C and lyophilized to remove water prior to extraction and analysis.

Before extraction, surrogate standards were added for determining recovery rates. The tissue samples were extracted three times via sonication in a 50 mL centrifuge tube with 40 mL of dichloromethane/acetone mixture (1:1, v/v), and all extracts were combined into a 60 mL glass tube and concentrated to 10 mL. An aliquot of 2 mL of this extract was removed for lipid analysis, while the remaining 8 mL was concentrated to near dryness and reconstituted in 1.0 mL of acetone–hexane (1:9, v/v). To remove residual lipids, the samples were filtered through a 2 g Florisil cartridge and eluted with 20 mL of the acetone–hexane mixture into a 20 mL glass test tube. The samples were then concentrated to 100 μL, and an internal standard was added prior to GC/MS analysis.^4^

The biota-sediment accumulation factor (BSAF) was calculated for each sample (eq 2)
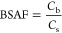
2where *C*_b_ is the concentration in the worm tissues normalized over the lipid content (ng/g, OC) and *C*_s_ is the sediment concentration normalized over the organic carbon content of the sediment (ng/g, OC).

### Instrumental Analysis

All samples were analyzed on an Agilent 6890N GC equipped with an Agilent 5975 mass spectrometry detector (MS or MSD) operating in the electron ionization (EI) mode for structural identification and quantification of the target analytes. All samples were injected (2 μL) into the GC at 200 °C in the splitless mode, and separation was achieved using a 30 m × 0.25 mm × 0.25 μm DB-5 fused silica capillary column (Agilent, Wilmington, DE). The initial oven temperature was set at 80 °C (held for 1 min), ramped to 210 °C at a rate of 10 °C/min, and then ramped once more to 300 °C at 5 °C/min and held for 5 min. The transfer line, ion source, and MS detector were set at 300, 230, and 150 °C, respectively. The carrier gas (helium, 99.999% purity) flow rate was 1.0 mL/min.

### Quality Assurance and Quality Control

During the study, several steps were taken to ensure quality control and integrity of analysis. All samples had three replicates, and laboratory blanks were included for PE film, sediment, and tissue analysis, in which no target analytes were detected. An external calibration curve was constructed using calibration standards prepared on the same day of analysis and was only used when the regression coefficients were ≥0.99. The recoveries of PCB 67 and PCB 191 ranged between 84.3 and 106% for all sample media. Limits of detection were set to three times the background noise and were determined to range from 0.05 to 0.1 ng/L.

Statistical significance and linear regression analysis were determined or calculated (i.e., via Pearson’s correlation coefficients, one-way Analysis of Variance (ANOVA), or Student’s *t*-tests) using SigmaPlot 12.0 or Prism 6 (Systat Software, San Jose, CA and GraphPad, San Diego, CA, respectively).

## Results and Discussion

### Carbonaceous Sorbent Amendment and *C*_free_

**Figure 1:**
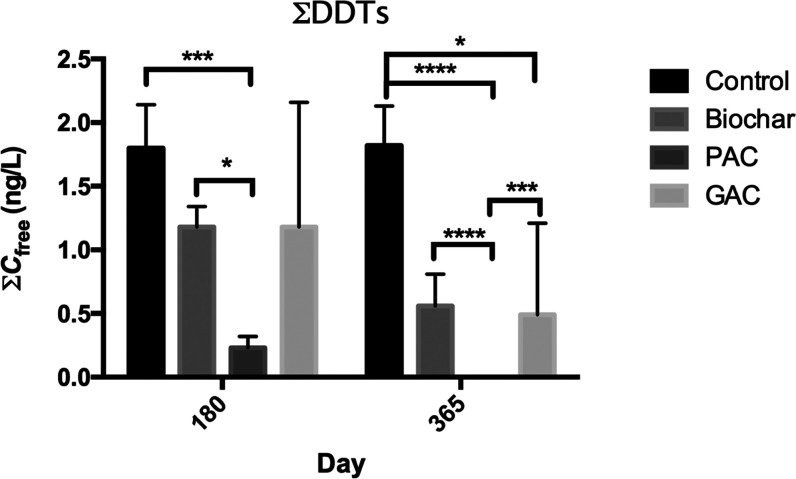
Sum of *C*_free_ (ng/L) in each treatment over time. Asterisks (*) refer to significant differences between treatments (*p < 0.05, **p < 0.01, ***p < 0.005, ****p < 0.001).

**Figure 2:**
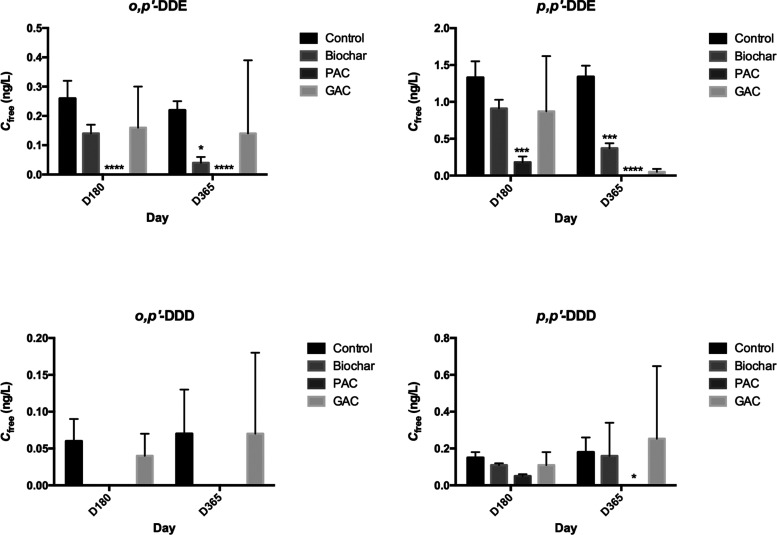
*C*_free_ (ng/L) of individual DDT compounds in each treatment over time. Asterisks (*) refer to significant differences between treatments (*p < 0.05, **p < 0.01, ***p < 0.005, ****p < 0.001).

Compared to the unamended control, the addition of carbonaceous materials to the contaminated sediment generally decreased the *C*_free_ of DDTs, although there were exceptions. For example, for ∑DDTs (*o*,*p*′- and *p*,*p*′-DDE, *o*,*p*′- and *p*,*p*′-DDD), the average values of *C*_free_ were 1.80 ± 0.34, 1.18 ± 0.16, 0.23 ± 0.09, and 1.18 ± 0.98 ng/L after 6 months of static incubation, and 1.82 ± 0.31, 0.56 ± 0.25, nd, and 0.49 ± 0.72 ng/L after 12 months of incubation, for the unamended control, biochar, PAC, and GAC-amended treatments, respectively (Figure 1, Figure 2). When ∑DDTs were considered, both biochar and PAC amendments resulted in a significant reduction (*p* < 0.05) in *C*_free_ as compared to the unamended control after 6 or 12 months of incubation. However, the GAC amendment did not lead to a significant reduction (*p* > 0.05) in *C*_free_ at the 6-month time point (Figure 1).

In the unamended sediment, *p*,*p*′-DDE exhibited the highest *C*_free_ values, followed by *o*,*p*′-DDE and *p*,*p*′-DDD, while *o*,*p*′-DDD was consistently found at the lowest concentration. The *C*_free_ values of the individual compounds corresponded to their bulk chemical concentrations in the sediment and were likely influenced further by their physicochemical properties such as hydrophobicity. The effectiveness of carbonaceous sorbent treatments appeared to be specific to the individual DDT compounds. Amendment with biochar or PAC significantly decreased the *C*_free_ of *o*,*p*′-DDE and *p*,*p*′-DDE after 6 or 12 months, with the relative reduction by PAC being greater than that by biochar. In comparison, the amendment of GAC did not result in appreciable decreases in *C*_free_ (Table 1; Figure 2). There was no consistent or significant effect of the carbonaceous sorbent amendment on the *C*_free_ of *o*,*p*′-DDD or *p*,*p*′-DDD (Table 1; Figure 2), likely due to their relatively lower hydrophobicity as compared to DDT or DDE. It must be also noted that the initial *C*_free_ values for *o*,*p*′-DDD were very small, and that the uncertainties in analysis likely also contributed to the lack of a discernible effect (Figure 2).

**Table 1:** *C*_free_ (ng/L) in Control (Unamended), Biochar, Powdered Activated Carbon (PAC), and Granular Activated Carbon (GAC) Amended Palos Verde Shelf Superfund Site Sediment at Different Sampling Times

Treatment	Day	*o*,*p*′-DDE	*p*,*p*′-DDE	*o*,*p*′-DDD	*p*,*p*′-DDD
Control	180	0.26 ± 0.06	1.33 ± 0.22	0.06 ± 0.03	0.15 ± 0.03
	365	0.22 ± 0.03	1.34 ± 0.15	0.07 ± 0.06	0.18 ± 0.08
Biochar	180	0.14 ± 0.03	0.91 ± 0.12	nd	0.11 ± 0.01
	365	0.04 ± 0.02	0.37 ± 0.07	nd	0.16 ± 0.18
PAC	180	nd	0.18 ± 0.08	nd	0.05 ± 0.01
	365	nd	nd	nd	nd
GAC	180	0.16 ± 0.14	0.87 ± 0.75	0.04 ± 0.03	0.11 ± 0.07
	365	0.14 ± 0.25	0.05 ± 0.04	0.07 ± 0.11	0.25 ± 0.39

The reductions in *C*_free_ due to the addition of carbonaceous sorbents in this study were consistent with the findings in some earlier studies. For example, powdered activated carbon and biochar decreased the *C*_free_ of DDTs in contaminated soil by >90%.^4^ Significant reductions in *C*_free_ in HOC-contaminated sediments after addition of carbonaceous materials were also reported in Chen et al.,^56^ Cornelissen et al.,^10^ Rakowska et al.,^19^ and Wang et al.^40^ The effect was typically attributed to the strong sorption capacity of the carbonaceous sorbents, and differences between different amendment materials were considered to be controlled by their physicochemical properties, such as specific surface areas.^57^ In this study, the specific surface areas (SSA) for the PAC and biochar were 706.2 and 690.4 m^2^/g, respectively. In Jia et al.,^55^ activated carbon had a much higher SSA than the biochar used in that study and was found to be more effective at reducing *C*_free_ of PBDEs in freshwater sediments. In contrast, the smaller effectiveness to GAC as compared to PAC in this study may be attributed to the steric effect and a slower DDT diffusion kinetics into the granulated form of activated carbon.

It is important to note that the sediment used in this study had a relatively high total organic carbon (TOC) content (5.7%). It can therefore be assumed that DDT was strongly sorbed to the sediment organic matter already before the addition of carbonaceous sorbents. For example, a study using a soil with a lower TOC showed a reduction in *C*_free_ of DDTs by 94–96% with the addition of AC at 0.2%, and a larger reduction at the 2% amendment rate.^4^ Beckingham and Ghosh^35^ found that amending sediments at rates closer to the native TOC content was more effective in reducing PCB bioaccumulation in benthic invertebrates. In another study using sediments with a higher TOC content (4.46%), GAC was found to be effective at reducing sediment porewater concentrations of PAHs only at a very high amendment rate (4%).^19^ The results from this and other studies suggested that the reduction in *C*_free_ after carbonaceous sorbent amendment depends on the ratio of the external carbon source over the native organic carbon, and limited effectiveness may be expected for organic carbon-rich sediments or soils.

### Effect of Carbonaceous Sorbent Amendment on Bioaccumulation

**Figure 3:**
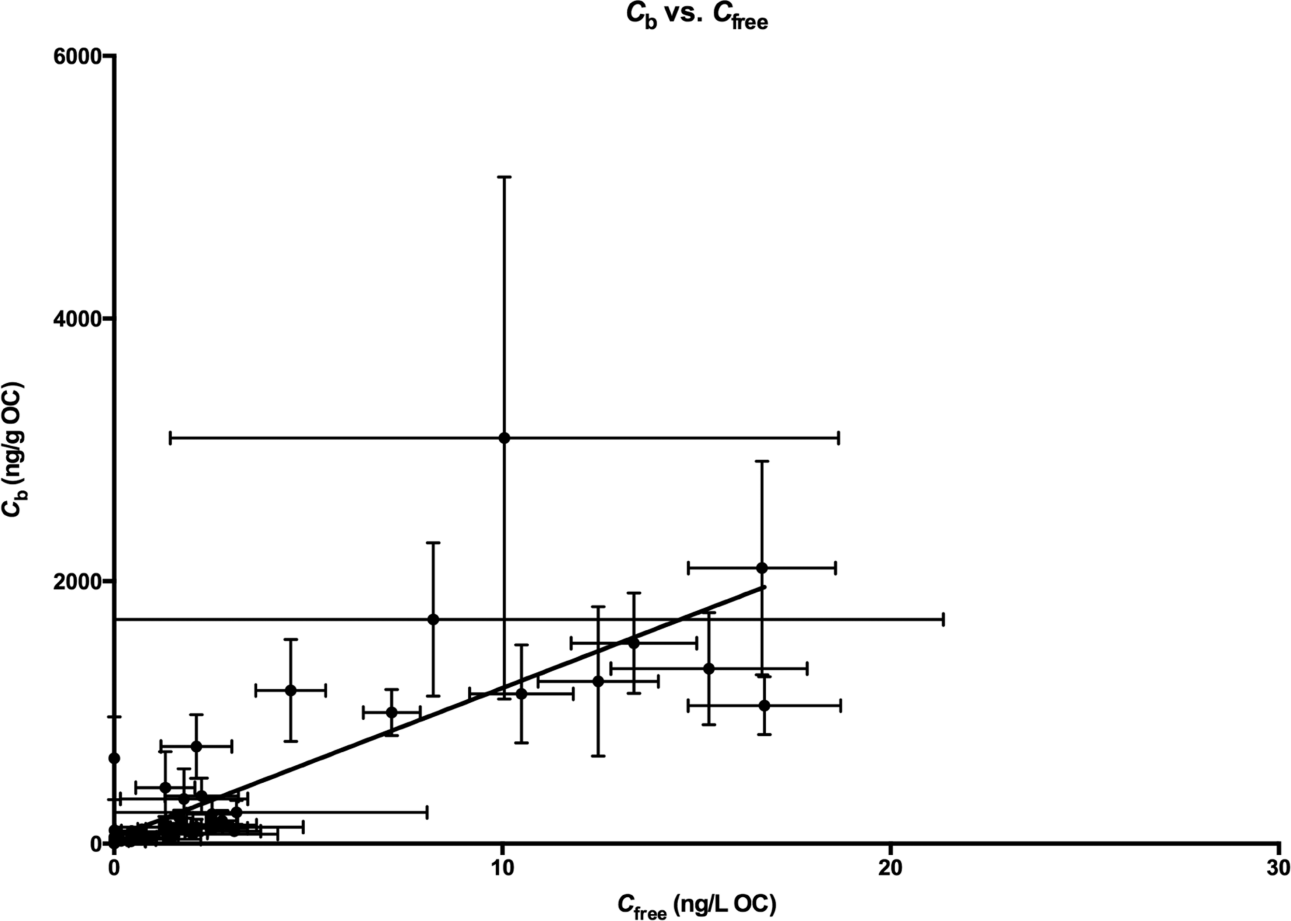
*C*_free_ (ng/L OC) versus *C*_b_ (ng/g OC). *R*^2^ = 0.66 and *p* < 0.0001.

As a direct measurement of bioavailability, residues of DDTs in *N. arenaceodentata* were concurrently derived after 96 h exposure. The exposure time was shorter than those generally recommended for bioaccumulation assays, and it was adopted to avoid ammonia toxicity originating from the sediment.^55^ Given that the test was carried out under the same conditions for all treatments, it may be assumed that the bioaccumulation by *N. arenaceodentata* should follow a similar kinetics, allowing an assessment of effects caused by carbonaceous sorbent amendment. The concentrations of DDTs in *N. arenaceodentata* were 130–290, 4600–12000, 190–320, and 960–1700 ng/g tissue (dw) for *o*,*p*′-DDE, *p*,*p*′-DDE, *o*,*p*′-DDD, and *p*,*p*′-DDD, respectively. The bioaccumulation generally followed patterns similar to those for *C*_free_, except for the GAC treatment. When *C*_free_ and *C*_b_ values of individual compounds from all treatments were plotted, there was a significant linear relationship between *C*_b_ and *C*_free_ (*R*^2^ = 0.66; *p* < 0.01) (Figure 3). However, even though the correlation was significant, it is apparent that the data were highly scattered, suggesting that factors other than *C*_free_ may also have affected the accumulation of DDTs in the benthic invertebrate. Considering that *N. arenaceodentata* is a deposit feeder, DDTs may be assimilated by the organism not only by dermal absorption but also by direct ingestion of contaminated sediment particles or carbonaceous sorbent particles. The results indicated that dermal uptake and particle ingestion were potentially significant sources for the accumulation of DDTs in *N. arenaceodentata* under the experimental conditions.

**Figure 4:**
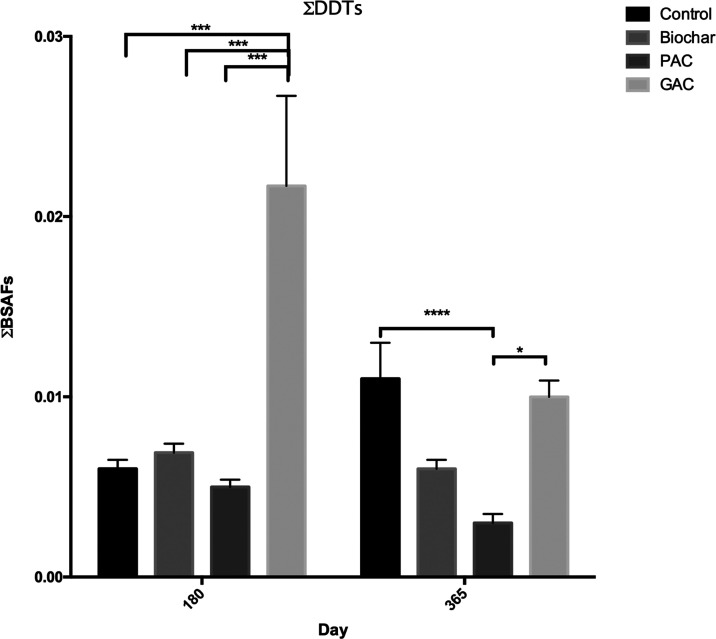
Biota-sediment accumulation factor (BSAF) values of different treatments at different sampling times. Asterisks (*) refer to significant differences between treatments (*p < 0.05, **p < 0.01, ***p < 0.005, ****p < 0.001).

**Figure 5:**
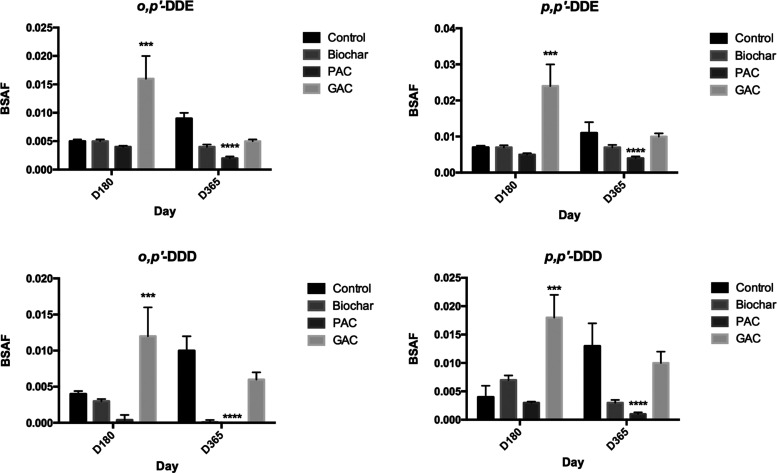
Biota-sediment accumulation factor (BSAF) values of DDT compounds in different treatments at different sampling times. Asterisks (*) refer to significant differences between treatments (*p < 0.05, **p < 0.01, ***p < 0.005, ****p < 0.001).

Apparent BSAF values were further calculated to assess the effectiveness of carbonaceous sorbent amendment in reducing bioaccumulation. The derived BSAF values were very small, ranging from 0.002 to 0.024, across all treatments, including the unamended sediment (Figure 4; Table 2). The amendment of carbonaceous sorbents did not result in a statistically significant reduction in BSAF (*p* > 0.05) as compared to the unamended control for most of the compounds and time points (Figure 5). A significant decrease in BSAF was observed for DDTs only in the PAC-amended sediment after 1 year of incubation (Table 2; Figure 4). No discernible decreasing trend was found for the rest of contaminant-carbonaceous sorbent combinations (Figure 5). In fact, after 180 days in the GAC-amended sediment, BSAFs increased unexpectedly. However, it must be noted again that the derived BSAFs were generally very small, which is not surprising, given the low *C*_free_ values, which in turn indicates that DDT had very limited bioavailability in the sediment after the extensive aging.^4,6^ The very small BSAFs may also be related to the fact that the exposure time in this study was only 4 days. In Wang et al. (2019),^5^ the same sediment was exposed to *Lumbriculus variegatus* for 28 days, and the resulting BSAFs for the unamended sediment were generally greater than those seen in this study, although they were also smaller than 0.05.

**Table 2:** Biota-Sediment Accumulation Factor (BSAF) Values in Control (Unamended), Biochar, Powdered Activated Carbon (PAC), and Granular Activated Carbon (GAC) Amended Palos Verde Shelf Superfund Site Sediment at Different Sampling Times

	Day	*o*,*p*′-DDE	*p*,*p*′-DDE	*o*,*p*′-DDD	*p*,*p*′-DDD
Control	180	0.005 ± 0.0003	0.007 ± 0.0005	0.004 ± 0.0004	0.004 ± 0.002
	365	0.009 ± 0.001	0.011 ± 0.003	0.010 ± 0.002	0.013 ± 0.004
Biochar	180	0.005 ± 0.0003	0.007 ± 0.0006	0.003 ± 0.0003	0.007 ± 0.0008
	365	0.004 ± 0.0004	0.007 ± 0.0007	0.0001 ± 0.0003	0.003 ± 0.0005
PAC	180	0.004 ± 0.0002	0.005 ± 0.0004	0.0004 ± 0.0007	0.003 ± 0.0002
	365	0.002 ± 0.0003	0.004 ± 0.0005	0	0.001 ± 0.0003
GAC	180	0.016 ± 0.004	0.024 ± 0.006	0.012 ± 0.004	0.018 ± 0.004
	365	0.005 ± 0.0003	0.010 ± 0.0009	0.006 ± 0.001	0.010 ± 0.002

The increases seen in bioaccumulation of DDTs in the GAC-amended sediment at 180 days were not anticipated. There have been studies suggesting that the changing microenvironment created by the addition of a carbonaceous sorbent may increase microbial activity or disturb sequestered contaminants, releasing previously sequestered HOCs.^58−60^ Another probable reason could be that *N. arenaceodentata* ingested some GAC granules during the exposure assay at this time interval, which could potentially contribute to the increased bioaccumulation. However, desorption of DDTs from the carbonaceous sorbents should be extremely slow, and the contribution, if any, may be negligible. It must be noted that the tested organism was very small in size (<1 cm) and that limited replications were used in the bioaccumulation experiments. These factors likely contributed to the uncertainties in the bioaccumulation observations. Other organisms, as well as HOCs and sediments (or soils) of different properties, should be considered in future studies to better characterize the significance of aging when considering the use of sorbent-based remediation strategies for historically contaminated sites.

### Limitations and Environmental Significance

Most soil or sediment sites contaminated by legacy contaminants such as DDT are somewhat similar to the PV Shelf Superfund site, where the initial contamination occurred decades ago, and the contaminants therefore have undergone prolonged aging in the environment. The extensive aging has often been found to result in reduced contaminant bioaccessibility or bioavailability.^5,6^ Although aging-induced reductions in contaminant availability have been increasingly acknowledged in the scientific community, this phenomenon has rarely been considered in the context of remediation practices. In this study, appreciable reductions in *C*_free_ or BSAFs after carbonaceous sorbent addition were not consistently achieved for a marine sediment contaminated with DDTs. These findings contradict observations in many studies to date that have considered the interactions of HOCs with external sorbents under various conditions.^55−62^ For example, in a study using spiked sediments, the uptake of PBDEs into a passive sampling device was reduced by 92–98% with the addition of only 0.5% of AC.^55^ In another study, a similar decrease in aqueous concentrations of DDTs was observed in a spiked sediment after the addition of AC or multiwalled carbon nanotubes.^61^ The use of freshly spiked sediments or soils, where the contaminant bioavailability is high due to the lack of aging, may have contributed to the pronounced effect. In studies where sediments with aged PCBs and PAHs were amended with AC under mixing conditions (to simulate tide wave disturbance), reductions in bioaccumulation or *C*_free_ were often observed.^14,26,27,29,30,33,57^ The differences between these studies and the current study may be attributed to different HOCs considered and/or experimental conditions used, such as the content and properties of indigenous organic carbon, and incubation conditions (e.g., mixing vs. static). For sediment beds at great depths and soil sites, little physical mixing is expected, and mass diffusion of contaminants may be slow. Mass transfer models have been successfully used to describe HOC distribution and bioaccumulation in contaminated sediments.^11,26,27,30,31,46,62^ It would be highly valuable to incorporate contaminant aging in the modeling approach to further refine such models and support their applications for the cleanup of contaminated sediment and soil sites.

It must be noted that bioavailability is an endpoint that is specific to the site and its ecosystem functions. While the native marine benthic invertebrate *N. arenaceodentata* was a suitable organism for assessing bioavailability of the Palos Verde Shelf Superfund site, as it serves as an important food source for bottom-feeding fish such as the California halibut (*P. californicus*), other nontarget organisms need to be selected to better predict the environmental risk of other compartments or sites. For example, for contaminated soil sites, soil invertebrates such as earthworms, as well as plants, may better reflect the bioavailability of contaminants. In addition, for contaminants such as DDTs, biomagnification through food chain and exposure to higher trophic level organisms, including humans, should be also considered. As shown in this and many other studies, chemically based measurements such as *C*_free_ are a good proxy for contaminant bioavailability and should be incorporated in the site assessment for remediation needs.

Observations from this study suggest that for historically contaminated sites, it is prudent to evaluate the need for, and value of, using carbonaceous or other sorbents by considering site- and contaminant-specific bioavailability before implementing actual mitigation practices. For contaminated sites where the contaminants have undergone extensive aging, as in the case of the PV Shelf Superfund site, aging, in combination with site-specific characteristics, may have already rendered the contaminants to be largely unavailable. Additional aging may be expected to further decrease the contaminant bioavailability, leading to further diminished environmental risks. In this context, the so-called monitored natural recovery (MNR) may be a better overall option to protect the environment while allowing time for the contaminated site to “self-clean” and recover. MNR requires the initial evaluation of a contaminated site for processes that will remove or sequester contaminants from a location without or in combination with other remediation techniques.^63^ These processes include contaminant burial or transport from a site, reductions in contaminant mobility (i.e., reductions in bioavailability due to strong sorption to sediment organic matter), and chemical or biological transformations.^63^ If these criteria are met, as is the case with the aged DDT residues at the PV Shelf, MNR can be a more cost-effective and environmentally friendly option. Partly for this reason and likely also due to the technical difficulties of other options presented by the great water depth (>80 m), MNR is in effect for the PV Shelf Superfund site.^49^ It is, therefore, crucial to determine the actual risks of historically contaminated sites before adopting a sorbent amendment-based remediation treatment.

## Supporting Information Available

Total concentrations (ng/g OC) of DDTs in the Palos Verdes sediment (Table S1); monitoring locations on the Palos Verdes Shelf (Figure S1); correlation of *C*_free_ (365 days)/*C*_free_ (0 day) and BSAF (365 days)/BSAF (0 day); *R*^2^ = 0.37, *p* = 0.015 (Figure S2); correlation of *C*_free_/*C*_s_ (365 days) over *C*_free_/*C*_s_ (0 day) and BSAF (365 days)/BSAF (0 day); *R*^2^ =0.49, *p* = 0.003 (Figure S3) (PDF)

## References

[ref1] Anderson M.; Conkle J.; Pacheco P.; Gan J. Delineation of organochlorine pesticide and PCB contamination in lake sediment by coupling hydroacoustic measurements with chemical analysis. Sci. Total Environ. 2013, 458–460, 117–124. 10.1016/j.scitotenv.2013.04.009. 23644565

[ref2] Bettinetti R.; Quadroni S.; Boggio E.; Galassi S. Recent DDT and PCB contamination in the sediment and biota of the Como Bay (Lake Como, Italy). Sci. Total Environ. 2016, 542, 404–410. 10.1016/j.scitotenv.2015.10.099. 26520265

[ref3] Zhang G.; Parker A.; House A.; Mai B. X.; Li X. D.; Kang Y. H.; Wang Z. S. Sedimentary records of DDT and HCH in the Pearl River Delta, South China. Environ. Sci. Technol. 2002, 36, 3671–3677. 10.1021/es0102888. 12322736

[ref4] Wang J.; Taylor A.; Xu C. Y.; Schlenk D.; Gan J. Evaluation of different methods for assessing bioavailability of DDT residues during soil remediation. Environ. Pollut. 2018, 238, 462–470. 10.1016/j.envpol.2018.02.082. 29587217

[ref5] Wang J.; Schlenk D.; Gan J. A direct method for quantifying the effects of aging on the bioavailability of legacy contaminants in soil and sediment. Environ. Sci. Technol. Lett. 2019, 6, 148–152. 10.1021/acs.estlett.8b00661.

[ref6] Taylor A. R.; Wang J.; Liao C.; Schlenk D.; Gan J. Effect of aging on bioaccessibility of DDTs and PCBs in marine sediment. Environ. Pollut. 2019, 245, 582–589. 10.1016/j.envpol.2018.10.126. 30471469 PMC6349416

[ref7] Lu Z.; Gan J.; Cui X.; Delgado-Moreno L.; Lin K. Understanding the bioavailability of pyrethroids in the aquatic environment using chemical approaches. Environ. Int. 2019, 129, 194–207. 10.1016/j.envint.2019.05.035. 31129496

[ref8] Cornelissen G.; Gustafsson O.; Bucheli T. D.; Jonker M. T. O.; Koelmans A. A.; Van Noort P. C. M. Extensive sorption of organic compounds to black carbon, coal, and kerogen in sediments and soils: Mechanisms and consequences for distribution, bioaccumulation, and biodegradation. Environ. Sci. Technol. 2005, 39, 6881–6895. 10.1021/es050191b. 16201609

[ref9] Cornelissen G.; Breedveld G. D.; Christanis K.; Kalaitzidis S.; Kibsgaard A.; Oen A. M. P. Strong sorption of native PAHs to pyrogenic and unburned carbonaceous geosorbents in sediments. Environ. Sci. Technol. 2006, 40, 1197–1203. 10.1021/es0520722. 16572775

[ref10] Cornelissen G.; Krusa M. E.; Breedveld G. D.; Eek E.; Oen A. P.; Arp H. P. H.; Raymond C.; Samuelsson G.; Hedman J. E.; Stokland O.; Gunnarsson J. S. Remediation of contaminated marine sediment using thin-layer capping with activated carbon- A field experiment in Trondheim Harbor, Norway. Environ. Sci. Technol. 2011, 45, 6110–6116. 10.1021/es2011397. 21671651

[ref11] Choi Y.; Cho Y. M.; Werner D.; Luthy R. G. *In situ* sequestration of hydrophobic organic contaminants in sediments under stagnant contact with activated carbon. 2. Mass transfer modeling. Environ. Sci. Technol. 2014, 48, 1843–1850. 10.1021/es404209v. 24410479

[ref12] Cornelissen G.; Amstaetter K.; Hauge A.; Schaanning M.; Beylich B.; Gunnarson J. S.; Breedveld G. D.; Oen A. P.; Eek E. Large-scale field study on thin-layer capping of Marine PCDD/F-contaminated sediments in Grenlandfjords, Norway: Physicochemical effects. Environ. Sci. Technol. 2012, 46, 12030–12037. 10.1021/es302431u. 23046183

[ref13] Ghosh U.; Luthy R. G.; Cornelissen G.; Werner D.; Menzie C. A. In-situ sorbent amendments: A new direction in contaminated sediment management. Environ. Sci. Technol. 2011, 45, 1163–1168. 10.1021/es102694h. 21247210 PMC3037809

[ref14] Werner D.; Higgins C. P.; Luthy R. G. The sequestration of PCBs in Lake Hartwell sediment with activated carbon. Water Res. 2005, 39, 2105–2113. 10.1016/j.watres.2005.03.019. 15922398

[ref15] Luthy R. G.; Aiken G. R.; Brusseau M. L.; Cunningham S. D.; Gschwend P. M.; Pignatello J. J.; Reinhard M.; Traina S. J.; Weber W. J.; Westall J. C. Sequestration of hydrophobic organic contaminants by geosorbents. Environ. Sci. Technol. 1997, 31, 3341–3347. 10.1021/es970512m.

[ref16] Goldberg E. Black Carbon in the Environment; John Wiley and Sons: New York, NY, 1985.

[ref17] Gustafsson Ö.; Haghseta F.; Chan C.; MacFarlane J.; Gschwend P. Quantification of the dilute sedimentary soot phase: implications for PAH speciation and bioavailability. Environ. Sci. Technol. 1997, 31, 203–209. 10.1021/es960317s.

[ref18] Patmont C. R.; Ghosh U.; LaRosa P.; Menzie C. A.; Luthy R. G.; Greenberg M. S.; Cornelissen G.; Eek E.; Collins J.; Hull J.; Hjartland T.; Glaza E.; Bleiler J.; Quadrini J. *In situ* sediment treatment using activated carbon: A demonstrated sediment cleanup technology. Integr. Environ. Assess. Manage. 2015, 11, 195–207. 10.1002/ieam.1589. PMC4409844 25323491

[ref19] Rakowska M. I.; Kupryianchyk D.; Grotenhuis T.; Rijnaarts H. H. M.; Koelmans A. A. Extraction of sediment-associated polycyclic aromatic hydrocarbons with granular activated carbon. Environ. Toxicol. Chem. 2013, 32, 304–311. 10.1002/etc.2066. 23147869

[ref20] Baldock J. A.; Smernik R. J. Chemical composition and bioavailability of thermally altered *Pinus resinosa* (red pine) wood. Org. Geochem. 2002, 33, 1093–1109. 10.1016/S0146-6380(02)00062-1.

[ref21] Lehmann J. A handful of carbon. Nature 2007, 447, 143–144. 10.1038/447143a. 17495905

[ref22] Ghosh U.; Luthy R. G.; Gillette J. S.; Zare R. N. Microscale location, characterization, and association of polycyclic aromatic hydrocarbons on harbor sediment particles. Environ. Sci. Technol. 2000, 34, 1729–1736. 10.1021/es991032t.

[ref23] National Research Council. Bioavailability of Contaminants in Soils and Sediments: Processes, Tools, and Applications; The National Academies Press: Washington, D.C. 2003.

[ref24] Abel S.; Akkanen J. Novel, activated carbon-based material for *in-situ* remediation of contaminated sediments. Environ. Sci. Technol. 2019, 53, 3217–3224. 10.1021/acs.est.8b06471. 30781950 PMC6727589

[ref25] Hale S. E.; Tomaszewski J. E.; Luthy R. G.; Werner D. Sorption of dichlorodiphenyltrichloroethane (DDT) and its metabolites by activated carbon in clean water and sediment slurries. Water Res. 2009, 43, 4336–4346. 10.1016/j.watres.2009.06.031. 19595428

[ref26] Hale S. E.; Werner D. Modeling the mass transfer of hydrophobic organic pollutants in briefly and continuously mixed sediment after amendment with activated carbon. Environ. Sci. Technol. 2010, 44, 3381–3387. 10.1021/es903582n. 20392086

[ref27] Janssen E. M. L.; Croteau M. N.; Luoma S. N.; Luthy R. G. Measurement and modeling of polychlorinated biphenyl accumulation from sediment for *Neanthes arenaceodentata* and response to sorbent amendment. Environ. Sci. Technol. 2010, 44, 2857–2863. 10.1021/es901632e. 20384377

[ref28] McLeod P. B.; Van Den Heuvel-Greve M. J.; Allen-King R. M.; Luoma S. N.; Luthy R. G. Effects of particulate carbonaceous matter on the bioavailability of benzo[a]pyrene and 2,2′,5,5′-tetrachlorobiphenyl to the clam, *Macoma balthica*. Environ. Sci. Technol. 2004, 38, 4549–4556. 10.1021/es049893b. 15461162

[ref29] Sun X.; Ghosh U. PCB bioavailability control in *Lumbriculus variegatus* through different modes of activated carbon addition to sediments. Environ. Sci. Technol. 2007, 41, 4774–4780. 10.1021/es062934e. 17695928

[ref30] Sun X.; Werner D.; Ghosh U. Modeling PCB mass transfer and bioaccumulation in a freshwater oligochaete before and after amendment of sediment with activated carbon. Environ. Sci. Technol. 2009, 43, 1115–1121. 10.1021/es801901q. 19320167

[ref31] Werner D.; Ghosh U.; Luthy R. G. Modeling polychlorinated biphenyl mass transfer after amendment of contaminated sediment with activated carbon. Environ. Sci. Technol. 2006, 40, 4211–4218. 10.1021/es052215k. 16856737

[ref32] Werner D.; Hale S. E.; Ghosh U.; Luthy R. G. Polychlorinated biphenyl sorption and availability in field-contaminated sediments. Environ. Sci. Technol. 2010, 44, 2809–2815. 10.1021/es902325t. 19961220 PMC2854002

[ref33] Zhou Y.; Miao D.; Gomez-Eyles J. L.; Ghosh U.; Bi M.; Li J.; Ren F. Comparative study on polychlorinated biphenyl sorption to activated carbon and biochar and the influence of natural organic matter. Chemosphere 2022, 287, 132239 10.1016/j.chemosphere.2021.132239. 34543896

[ref34] Abel S.; Akkanen J. A combined field and laboratory study on activated carbon-based thin layer capping in a PCB-contaminated boreal lake. Environ. Sci. Technol. 2018, 52, 4702–4710. 10.1021/acs.est.7b05114. 29606006 PMC6150667

[ref35] Beckingham B.; Ghosh U. Polyoxymethylene passive samplers to monitor changes in bioavailability and flux after activated carbon amendment to sediment in the field. Chemosphere 2013, 9, 1401–1407. 10.1016/j.chemosphere.2012.12.074. 23415491

[ref36] Sanders J. P.; Andrade N. A.; Menzie C. A.; Amos C. B.; Gilmour C. C.; Henry E. A.; Brown S. S.; Ghosh U. Persistent reductions in the bioavailability of PCBs at a tidally inundated Phragmites australis marsh amended with activated carbon. Environ. Toxicol. Chem. 2018, 37, 2496–2505. 10.1002/etc.4186. 29870109

[ref37] Cao X.; Ma L.; Liang Y.; Gao B.; Harris W. Simultaneous immobilization of lead and atrazine in contaminated soils using dairy-manure biochar. Environ. Sci. Technol. 2011, 45, 4884–4889. 10.1021/es103752u. 21542567

[ref38] Fu H.; Wei C.; Qu X.; Li H.; Zhu D. Strong binding of apolar hydrophobic organic contaminants by dissolved black carbon released from biochar: A mechanism of pseudomicelle partition and environmental implications. Environ. Pollut. 2018, 232, 402–410. 10.1016/j.envpol.2017.09.053. 28966024

[ref39] Kang S.; Jung J.; Choe J. K.; Ok Y. S.; Choi Y. Effect of biochar particle size on hydrophobic organic compound sorption kinetics: Applicability of using representative size. Sci. Total Environ. 2018, 619–620, 410–418. 10.1016/j.scitotenv.2017.11.129. 29156262

[ref40] Wang F.; Bu Q.; Xia X.; Shen M. Contrasting effects of black carbon amendments on PAH bioaccumulation by *Chironomus plumosus* larvae in two distinct sediments: role of water absorption and particle ingestion. Environ. Pollut. 2011, 159, 1905–1913. 10.1016/j.envpol.2011.03.033. 21531490

[ref41] Ahmad R.; Kookana R. S.; Megharaj M.; Alston A. M. Aging reduces the bioavailability of even a weakly sorbed pesticide (carbaryl) in soil. Environ. Toxicol. Chem. 2004, 23, 2084–2089. 10.1897/03-569. 15378982

[ref42] Gomez-Eyles J. L.; Yupanqui C.; Beckingham B.; Riedel G.; Gilmour C.; Ghosh U. Evaluation of biochars and activated carbons for *in situ* remediation of sediments impacted with organics, mercury, and methylmercury. Environ. Sci. Technol. 2013, 47, 13721–13729. 10.1021/es403712q. 24168448

[ref43] Lehmann J.; Cowie A.; Masiello C. A.; Kammann C.; Woolf D.; Amonette J. E.; Cayuela M. L.; Camps-Arbestain M.; Whitman T. Biochar in climate change mitigation. Nat. Geosci. 2021, 14, 883–892. 10.1038/s41561-021-00852-8.

[ref44] Sparrevik M.; Saloranta T.; Cornelissen G.; Eek E.; Fet A.; Breedveld G.; Linkov I. Use of life cycle assessments to evaluate the environmental footprint of contaminated sediment remediation. Environ. Sci. Technol. 2011, 45, 4235–4241. 10.1021/es103925u. 21520943

[ref45] Cheng G.; Liu H.; Dong T.; Li Q.; Sun M.; Lou L. Assessment and prediction of the effect of ageing on the adsorption of nonylphenol in black carbon-sediment systems. J. Environ. Sci. 2021, 102, 216–225. 10.1016/j.jes.2020.09.008. 33637246

[ref46] Thompson J. M.; Hsieh C. H.; Hoelen T. P.; Weston D. P.; Luthy R. G. Measuring and modeling organochlorine pesticide response to activated carbon amendment in tidal sediment mesocosms. Environ. Sci. Technol. 2016, 50, 4769–4777. 10.1021/acs.est.5b05669. 27040592

[ref47] Umeh A. C.; Duan L.; Naidu R.; Semple K. T. Time-dependent remobilization of nonextractable benzo[a]pyrene residues in contrasting soils: Effects of aging, spiked concentration, and soil properties. Environ. Sci. Technol. 2018, 52, 12295–12305. 10.1021/acs.est.8b03008. 30351040

[ref48] Eganhouse R. P.; Pontolillo J. DDE in sediments of the Palos Verdes Shelf, California: in situ transformation rates and geochemical fate. Environ. Sci. Technol. 2008, 42, 6392–6398. 10.1021/es7029619. 18800506

[ref49] U.S. Environmental Protection Agency. Revised final data report for the fall 2009 sediment sampling program: Palos Verdes Shelf (OU 5 of the Montrose Chemical Corporation Superfund Site) Los Angeles County, CA. Region IX; United States Environmental Protection Agency: San Francisco, CA, 2013.

[ref50] U. S. Environmental Protection Agency. Interim record of decision: Palos Verdes Shelf operable unit 5 of Montrose Chemical Corporation Superfund Site, Los Angeles County, CA; Region IX; United States Environmental Protection Agency: San Francisco, CA, 2009.

[ref51] Liao C. Y.; Taylor A.; Tang C. L.; Gully J. R.; Kenney W. F.; Brenner M.; Gan J. Historical record and flux of DDTs and PCBs to the Palos Verdes Shelf Superfund Site, California. Sci. Total Environ. 2017, 581, 697–704. 10.1016/j.scitotenv.2016.12.182. 28082055

[ref52] Fernandez L. A.; Lao W.; Maruya K. A.; White C.; Burgess R. M. Passive sampling to measure baseline dissolved persistent organic pollutant concentrations in the water column of the Palos Verdes Shelf Superfund site. Environ. Sci. Technol. 2012, 46, 11937–11947. 10.1021/es302139y. 23062073

[ref53] Fernandez L. A.; Lao W.; Maruya K. A.; Burgess R. M. Calculating the diffusive flux of persistent organic pollutants between sediments and the water column on the Palos Verdes Shelf Superfund Site using polymeric passive samplers. Environ. Sci. Technol. 2014, 48, 3925–3934. 10.1021/es404475c. 24564763

[ref54] Anderson B. S.; Hunt J. W.; Phillips B. M.; Tudor S.; Fairey R.; Newman J.; Puckett H. M.; Stephenson M.; Long E. R.; Tjeerdema R. S. Comparison of marine sediment toxicity test protocols for the amphipod *Rhepoxynius abronius* and the polychaete worm *Nereis (Neanthes) arenaceodentata*. Environ. Toxicol. Chem. 1998, 17, 859–866. 10.1002/etc.5620170513.

[ref55] Jia F.; Gan J. Comparing black carbon types in sequestering polybrominated diphenyl ethers (PBDEs) in sediments. Environ. Pollut. 2014, 184, 131–137. 10.1016/j.envpol.2013.08.009. 24047549 PMC3915297

[ref56] Chen Y.; Yu W.; Zheng R.; Li J.-Y.; Zhang L.; Wang Q.; Yin J.; Jin L. Magnetic activated carbon (MAC) mitigates contaminant bioavailability in farm pond sediment and dietary risks in aquaculture products. Sci. Total Environ. 2020, 736, 139185 10.1016/j.scitotenv.2020.139185. 32485365

[ref57] Millward R. N.; Bridges T. S.; Ghosh U.; Luthy R. G.; Zimmerman J. R. Addition of activated carbon to sediments to reduce PCB bioaccumulation by the polychaete, *Neanthes arenaceodentata*, and the amphipod, *Leptocheirus plumulosus*. Environ. Sci. Technol. 2005, 39, 2880–2887. 10.1021/es048768x. 15884389

[ref58] Gregory S. J.; Anderson C. W. N.; Camps-Arbestain M.; Biggs P. J.; Ganley A. R. D.; O’Sullivan J. M.; McManus M. T. Biochar in co-contaminated soil manipulates arsenic solubility and microbial community structure, and promotes organochlorine degradation. PLoS One 2015, 10, 0125393 10.1371/journal.pone.0125393. PMC4414470 25923541

[ref59] Kjellerup B. V.; Naff C.; Edwards S. J.; Ghosh U.; Baker J. E.; Sowers K. R. Effects of activated carbon on reductive dechlorination of PCBs by organohalide respiring bacteria indigenous to sediments. Water Res. 2014, 52, 1–10. 10.1016/j.watres.2013.12.030. 24440760

[ref60] Pignatello J. J.; Mitch W. A.; Xu W. Activity and reactivity of pyrogenic carbonaceous matter toward organic compounds. Environ. Sci. Technol. 2017, 51, 8893–8908. 10.1021/acs.est.7b01088. 28753285

[ref61] Hua S.; Gong J. L.; Zeng G. M.; Yao F. B.; Guo M.; Ou X. M. Remediation of organochlorine pesticides contaminated lake sediment using activated carbon and carbon nanotubes. Chemosphere 2017, 177, 65–76. 10.1016/j.chemosphere.2017.02.133. 28284117

[ref62] Choi Y.; Cho Y. M.; Luthy R. G.; Werner D. Predicted effectiveness of in-situ activated carbon amendment for field sediment sites with variable site- and compound-specific characteristics. J. Hazard. Mater. 2016, 301, 424–432. 10.1016/j.jhazmat.2015.09.016. 26410271

[ref63] Magar V. S.; Wenning R. J. The role of monitored natural recovery in sediment remediation. Integr. Environ. Assess. Manage. 2006, 2, 66–74. 10.1002/ieam.5630020112. 16640320

