# Supplementary material for: Limited Effectiveness of Carbonaceous Sorbents in Sequestering Aged Organic Contaminants in Sediments
Source: Environ Sci Technol. 2023 Jun 15;57(25):9385–93. doi: 10.1021/acs.est.3c02309 (PMC10308819; doi:10.1021/acs.est.3c02309)
Supplement: Supplementary file 1 — es3c02309_si_001.pdf [file es3c02309_si_001.pdf]

Supplemental Information

Limited Effectiveness of Carbonaceous Sorbents in Sequestering Aged Organic Contaminants in Sediment

Allison R. Taylor,[†] Jie Wang,[†,‡] Parminder Kaur,[†] Daniel Schlenk,[†] Jay Gan[†,*]

[†]Department of Environmental Sciences, University of California, Riverside, California 92521, United States

[‡]College of Resources and Environmental Sciences, China Agricultural University, Beijing 100193, China

*Corresponding author: Jay Gan

Department of Environmental Sciences,

University of California, Riverside, CA 92521

Tel: (951) 827-2712

E-mail: jgan@ucr.edu

Table S1. Total concentrations (ng/g OC) of DDTs in the Palos Verdes sediment.

Treatment	*o,p'*-DDE	*p,p'*-DDE	*o,p'*-DDD	*p,p'*-DDD
C	24700 ± 5240	173000 ± 53000	6880 ± 2500	32400 ± 21300
BC	23200 ± 4210	150000 ± 31400	6880 ± 2060	20500 ± 4880
PAC	23800 ± 4420	150000 ± 30400	6050 ± 1760	23400 ± 13700
GAC	23900 ± 3520	147000 ± 22400	6380 ± 1130	23300 ± 6340

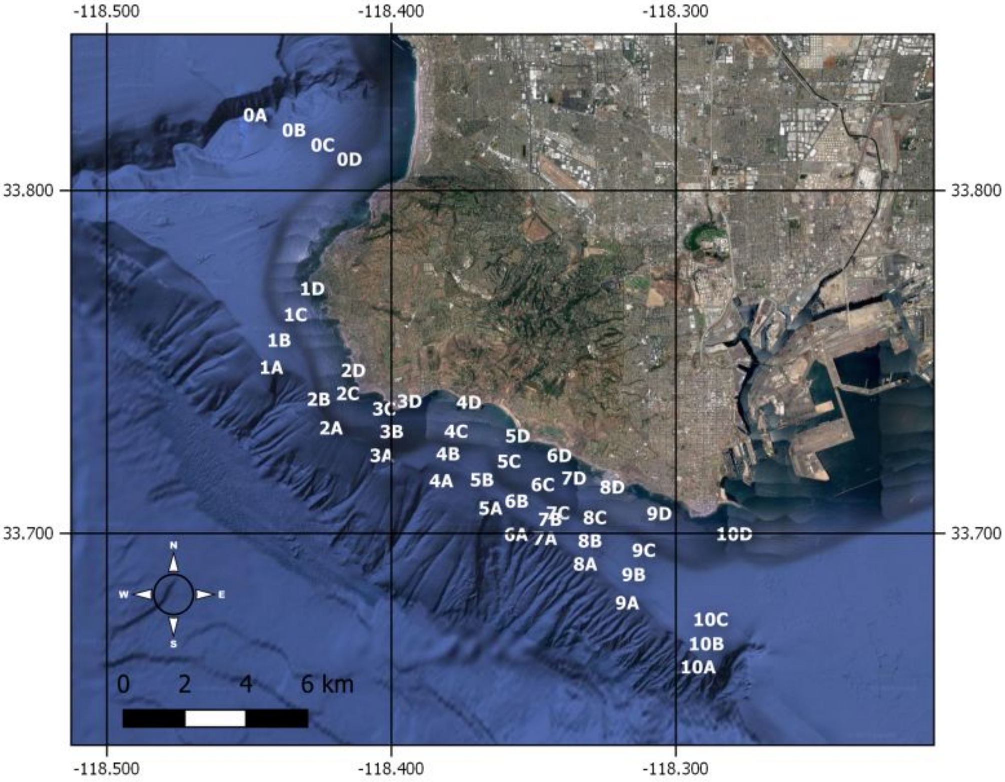

Figure S1. Monitoring locations on the Palos Verdes Shelf. 8C is located at the sewer system outfall and is the sampling location used in this study.

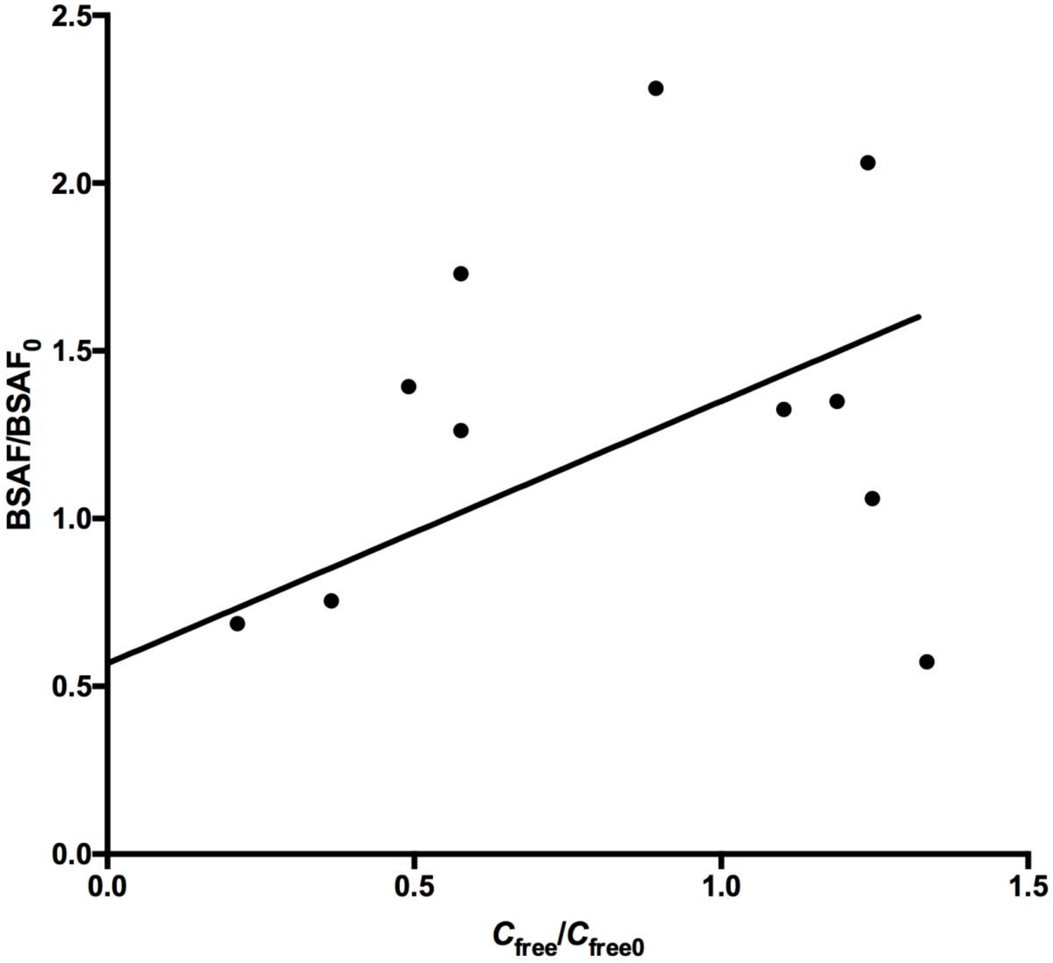

Figure S2. Correlation of C_{free} (365 d)/C_{free} (0 d) and BSAF (365 d)/BSAF (0 d); $R^2 = 0.37$, $p = 0.015$.

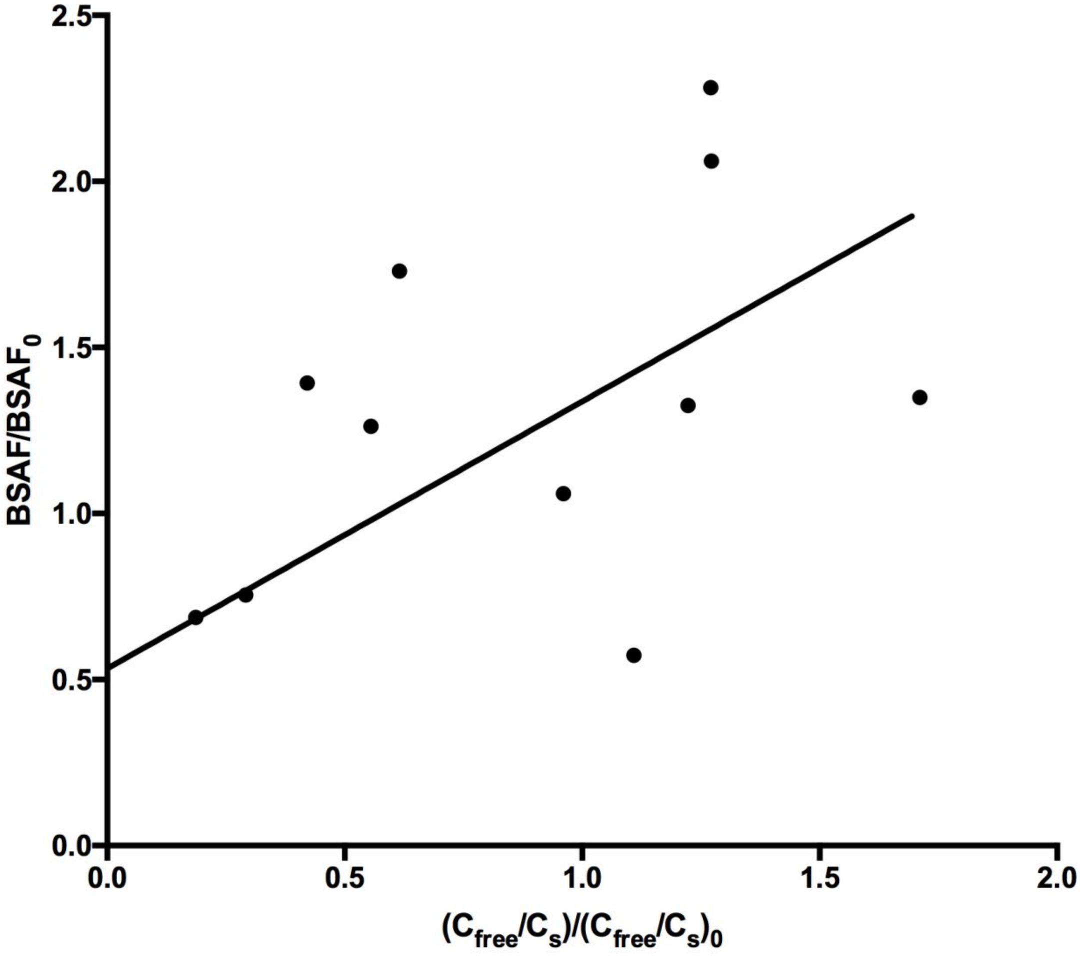

Figure S3. Correlation of C_{free}/C_s (365 d) over C_{free}/C_s (0 d) and BSAF (365 d)/BSAF (0 d); $R^2 = 0.49$, $p = 0.003$.
